# Step cadence to guide physical activity intensity in coronary heart disease

**DOI:** 10.3389/fspor.2026.1763343

**Published:** 2026-03-16

**Authors:** Amanda Lönn, Brad Clark, Theo Niyonsenga, Kate Pumpa, Maria Bäck, Arjun Rangaraj, Tze Hao Wong, Soraya Joseph, Ahmed Khan, Peter Scott, Rachel Davey, Nicole Freene

**Affiliations:** 1 Health Research Institute, University of Canberra, Bruce, ACT, Australia; 2 Department of Physical Activity and Health, The Swedish School of Sport and Health Sciences (GIH), Stockholm, Sweden; 3 Research Institute for Sport and Exercise, University of Canberra, Bruce, ACT, Australia; 4 Institute for Sport and Health, University College Dublin, Dublin, Ireland; 5 Department of Occupational Therapy and Physiotherapy, Sahlgrenska University Hospital, Gothenburg, Sweden; 6 Department of Health, Medicine and Caring Sciences, Unit of Physiotherapy, Linköping University, Linköping, Sweden; 7 Canberra Health Services, Garran, ACT, Australia; 8 National Capital Private Hospital, Garran, ACT, Australia; 9 North Canberra Hospital, Bruce, ACT, Australia; 10 Heart of Canberra, Deakin, ACT, Australia

**Keywords:** coronary heart disease, physical activity, stepping rate, walking, wearable devices

## Abstract

**Introduction:**

Regular physical activity at a moderate-to-vigorous intensity is associated with a lower risk of recurrent cardiovascular events and mortality in individuals with coronary heart disease (CHD). Walking is a common form of physical activity; however, there is no evidence-based recommendation for the optimal step cadence (steps per minute) to achieve these intensity levels among individuals with coronary heart disease. Thus, this study aimed to establish the agreement between manually counted step cadence and step cadence assessed by waist- and wrist-worn accelerometers during treadmill walking among individuals with CHD. Next, the study aimed to establish the association between manually counted and accelerometer-assessed step cadence with cardiorespiratory intensity, followed by the development and validation of cadence thresholds for moderate- and vigorous-intensity levels.

**Methods:**

Participants (*n* = 87) performed a graded treadmill test. Step cadence was counted manually and by a waist- and wrist-worn accelerometer (Actigraph GT3x). Step cadence and oxygen consumption were measured during the final minute of walking at four speeds (3–6 km·h^−^^1^). Agreement between manual and accelerometer cadence was established using Bland–Altman analyses. Step cadence thresholds for relative (%VO₂ peak) and absolute (METs) intensity were derived using generalized estimating equations in a training cohort (70%) and validated in a validation cohort (30%).

**Results:**

Accelerometers underestimated the aggregated cadence compared with manual assessments, with a mean difference of −16 and −31 steps/min for waist- and wrist-worn accelerometers, respectively, with a higher level of agreement at higher speeds. Manually assessed cadence thresholds for moderate intensity were 99 (relative) and 92 (absolute) steps/min compared with 61 and 45 steps/min from waist-worn accelerometers. For vigorous intensity, manual and accelerometer thresholds were similar: 116 vs. 112 (relative) and 126 vs. 135 (absolute) steps/min. Both methods effectively identified valid thresholds for moderate and vigorous cardiorespiratory intensity.

**Conclusion:**

Step cadence is a valid indicator of intensity in CHD populations, with manual and accelerometer methods showing comparable accuracy, despite accelerometer underestimation at lower speeds.

**Clinical Trial Registration:**

Australian New Zealand Clinical Trials Registry (ANZCTR): ACTRN12623000605695 (02062023).

## 1 Introduction

Physical activity at a moderate-to-vigorous intensity (MVPA) is recommended for secondary prevention among individuals with coronary heart disease (CHD) (1, 2). In the adult general population, walking is the most common form of physical activity, integrated into daily living, exercise, transport, and work (3). A step serves as the fundamental unit of walking (4), step count measures volume of physical activity, while step cadence (steps/minute) reflects its intensity. Both step volume and cadence are independently associated with a lower risk of premature all-cause mortality in the general population (5).

In recent decades, technological development has led to the widespread use of activity monitors for step detection in both clinical and general populations (6–8). The 2018 Physical Activity Guidelines Advisory Committee emphasized the importance of evaluating the accuracy of step cadence derived from activity monitors (3). Previous studies have concluded that activity monitors commonly underestimate step cadence compared with manual assessment, irrespective of the type or placement of the activity monitor (9–11). However, there is a lack of evidence regarding the validity of step cadence for activity monitors in individuals from different age groups or clinical populations.

Studies in the general adult population have defined step cadence thresholds for MVPA. For example, a study in middle-aged adults found a threshold of 80 steps/min assessed with waist-worn accelerometers in free-living conditions for moderate intensity (12). In contrast, studies from laboratory settings, with manually assessed steps, found a higher threshold, ranging from 100 to 110 steps/min, for moderate intensity among individuals ≥40 years. For vigorous intensity, the threshold varied between 115 and 130 steps/minute (13–15). Step cadence thresholds for both moderate and vigorous intensity decline with increasing age when established in association with relative cardiorespiratory intensity (13).

While step cadence thresholds for MVPA have been established in the general population, no such thresholds currently exist for individuals with CHD. Considering the reduced cardiorespiratory fitness of this population (16) relative to the general population (17), cadence thresholds may differ significantly and should be explored based on both manual and accelerometer assessments. Improved knowledge of accurate step cadence assessment and its association with cardiorespiratory intensity may contribute to guidelines for prescribing walking intensity for individuals with CHD. Thus, this study aimed to establish agreement between manually counted step cadence and step cadence assessed by waist- and wrist-worn accelerometers during treadmill walking among individuals with CHD. Next, we aimed to establish the association between manually counted and accelerometer-assessed step cadence with relative (percentage of VO_2_ peak) and absolute (metabolic equivalents) cardiorespiratory intensity, followed by the development and validation of cadence thresholds for moderate- and vigorous-intensity levels.

## 2 Materials and methods

A cross-sectional validation study exploring step cadence in people with CHD was conducted between August 2023 and December 2024.

### 2.1 Participants

Using voluntary sampling, participants were recruited through healthcare professionals at cardiology clinics, phase II cardiac rehabilitation programs, and via media advertisements in the Australian Capital Territory. Eligibility criteria included age ≥18 years, stable CHD, English-language proficiency, sufficient cognitive capacity to engage in the research process, and medical clearance from a cardiologist or general practitioner. Exclusion criteria included primary diagnosis of atrial fibrillation, New York Heart Association class III-IV symptoms of heart failure, uncontrolled arrhythmias, unstable angina, severe chronic obstructive pulmonary disease, uncontrolled hypertension, symptomatic peripheral artery disease, uncontrolled diabetes, or inability to perform a maximal walking test on a treadmill. Demographic and clinical information were collected using a questionnaire, including sex, age, type and date of CHD event/procedure, cardiac medications, smoking habits, physical activity levels, and other medical conditions.

### 2.2 Measurement

Height was measured to the nearest 0.5 cm using a stadiometer (SECA, GmbH & Co Hamburg, Germany). Body mass was measured to the nearest 0.1 kg using a calibrated scale (SECA GmbH & Co. KG, Hamburg, Germany), with participants barefoot and without heavy outer clothing. Body mass index (BMI) was calculated as the body mass (kg) divided by the height squared (m^2^). Participants were fitted with a 12-lead electrocardiogram (ECG, Norav Medical Inc. Delray Beach, FL, USA) to monitor heart rate throughout the test session. Actigraph accelerometers (wGT3X-BT, Pensacola, Florida, USA) were worn at two sites: secured in an elastic belt around the waist (placed over the right hip) and using a wristband on the non-dominant wrist. Expired gases were collected via a one-way valve (Hans Rudolph Inc., Shawnee, KS, USA) and analyzed using a stationary metabolic gas analyzer (TrueOne, Parvomedics, Sandy, UT, USA).

### 2.3 Testing procedure

The study protocol required participants to take their usual medications, refrain from eating for 3 h before the test, and avoid exercise and caffeine on the day of the test. Participants arrived by taxi to be as rested as possible. A medical assessment was completed on the day prior to testing. In the laboratory, the participants initially lay supine for 25 min to measure their resting metabolic rate (RMR). The average VO_2_ consumption between the 10th and 20th minutes was used to calculate RMR, with extreme values excluded. Then, participants performed a graded walking treadmill test under the supervision of trained staff. Participants were encouraged not to use the handrail during the test, but if they did, they used the arm without the wrist accelerometer. The treadmill test consisted of two parts. Part 1 included four 5-min stages at 3, 4, 5, and 6 km·h^−^^1^. During the final minute of each stage, cadence (steps/min) was video recorded and manually tallied (ground truth) using a hand counter, alongside accelerometer assessment from the waist and wrist. In addition, VO_2_ consumption was assessed simultaneously from indirect calorimetry. From the second part of the treadmill test, the speed remained constant (6 km·h^−^^1^), but the treadmill gradient increased by 3.5% for every minute, aiming to reach the VO_2_ peak. The test could be stopped at any point on the participant's request or if the research staff identified safety concerns. To be considered as having reached VO₂ max, participants had to meet at least one of the following criteria: a respiratory exchange ratio (RER) >1.10 or a heart rate within −10% of the age-predicted maximum (220-age).

### 2.4 Data processing

Accelerometer data were processed using Actilife software, version 6.13.4. Data were collected triaxially at a sampling rate of 30 Hz and downloaded as 1-second epochs. A normal frequency filter was applied. Peak VO_2_ was calculated as the highest 30-s average during the graded treadmill test. Absolute intensity—metabolic equivalents (METs)—was calculated as the individual's average VO_2_ divided by their RMR. Relative intensity was calculated as a percentage of peak VO_2_.

### 2.5 Power analysis

An *a priori* power analysis for linear regression (with one predictor, medium effect size *f*^2^ = 0.15, *α* = 0.05, and power = 0.80) indicated that a minimum total sample size of 55 participants was required.

### 2.6 Statistical analyses

Analyses were performed in R studio, version 2023.03.1. The normality of the variables was assessed through visualization, the Shapiro–Wilk test, and examination of skewness and kurtosis. Differences in descriptive characteristics for subgroups (by mean age and validation vs. training cohort) were examined using chi-square tests for categorical variables, Mann–Whitney *U*-tests for non-parametric and unpaired *t*-tests for parametric continuous variables. The significance level was set at *p* < 0.05. The level of agreement between the manual and accelerometer-assessed step cadence was evaluated using the Bland–Altman method, with mean differences and 95% limits of agreement (18). Linear regression was used to assess whether the mean difference and limits of agreement varied across average values of accelerometer and manually assessed step cadence.

To explore the correlation between aggregate step cadence and relative and absolute cardiorespiratory intensity, repeated measures correlation (R library, rmcorr) was used (19). The correlation was interpreted as weak (*r* < 0.10), modest (*r* = 0.1–0.29), moderate (*r* = 0.3–0.49), strong (*r* = 0.5–0.79), or very strong (*r* = 0.8–1.0) (20).

Training and validation cohorts were created using a stratified random split of the full sample with the createDataPartition function from the caret package in R for model development and validation of step cadence. Then, to examine the association between manual and accelerometer step cadence with absolute and relative intensity, linear generalized estimating equations (GEEs) were performed in the training cohort (R library *geepack*) (21). Both unadjusted models and models adjusted for covariates potentially affecting the association—age, sex, BMI, and height—were analyzed, with participant ID included as a unique identifier. Step cadence thresholds of moderate and vigorous intensity were calculated by rearranging the unadjusted GEE linear regression equations for step cadence (independent variable) with relative and absolute cardiorespiratory intensity (dependent variable). In these models, the step cadence was kept continuous, and the absolute and relative cardiorespiratory intensities were used as categorical variables, using established thresholds of absolute and relative cardiorespiratory intensity based on American College of Sports Medicine (ACSM) criteria (17). For relative intensity, the levels were moderate (≥46% VO_2peak_) and vigorous (≥64% VO_2peak_). For absolute intensity, the levels were moderate (≥3.0 METs) and vigorous (≥ 6.0 METs) (17). Finally, the developed step cadence thresholds were validated in the validation cohort by estimating associated sensitivity, specificity, positive predictive values (PPV), negative predictive values (NPV), and balanced accuracy (the average of sensitivity and specificity, providing equal weight to both classes).

To establish potential differences in step cadence agreement across age groups, Sensitivity analyses were conducted to assess potential differences in step cadence agreement between participants below the mean age and those at or above the mean age An interaction term (age group × step cadence) was added for the GEE analyses, with *p* < 0.05 indicating differences between age groups.

## 3 Results

A total of 170 individuals were screened; of these, 87 met the inclusion criteria and completed both the RMR and treadmill tests (Supplementary Appendix Figure A1). Participant characteristics are presented in Table 1. The mean age was 66 years, with the majority being men (87%). Most participants had undergone percutaneous coronary interventions (PCI, 71%) and had experienced their most recent cardiac event or coronary intervention a median of 1.4 years before study enrolment. All participants were non-smokers, 9% had a heart failure diagnosis (*n* = 6 NYHA classification I, *n* = 2 NYHA classification II), 9% had type two diabetes, and 34% had other comorbidities (e.g., cancer, osteoarthritis). Participants self-reported a median of 240 min/week of MVPA, and the mean peak VO_2_ was 24 mL/kg/min during the treadmill test. There were 33% who achieved RER ≥1.10, 52% reached 90% or more of their predicted heart rate max, and 29% met both criteria. None of the participants had angina symptoms or arrhythmias during the test session. The validation cohort reported significantly higher self-reported physical activity compared with the training cohort (Table 1). There were significant differences between age groups: Older individuals (≥66 years) were more likely to be male, had lower RMR and peak VO_2_, and fewer achieved an RER of 1.10 (Supplementary Appendix Table A1).

**Table 1:** Participant characteristics, total, training, and validation cohorts.

Characteristic	Total cohort (*n* = 87)	Training cohort (*n* = 64)	Validation cohort (*n* = 23)
Age (years), mean (SD)	66 (10)	66 (10)	66 (11)
Male, *n* (%)	76 (87%)	58 (91%)	18 (78%)
Height (m), mean (SD)	1.74 (0.08)	1.75 (0.08)	1.72 (0.09)
Weight (kg), mean (SD)	83 (13)	84 (13)	80 (15)
BMI, mean (SD)	27.5 (3.9)	27.6 (3.5)	27.2 (5.0)
Coronary treatment/intervention, *n* (%)			
Coronary artery bypass graft (CABG)	17 (20%)	13 (20%)	4 (17%)
Conservative treatment	8 (9.2%)	4 (6.3%)	4 (17%)
Percutaneous coronary intervention (PCI)	62 (71%)	47 (73%)	15 (65%)
Days from last diagnosis/intervention, median (IQR)	515 (1,228)	557 (1,156)	382 (1,439)
Beta blockers, *n* (%)	28 (35%)	19 (33%)	9 (39%)
Moderate to vigorous physical activity (min/week), median (IQR)	240 (270)	207 (180)	315 (210)*
Resting metabolic rate (mL/kg/min), mean (SD)	2.83 (0.47)	2.83 (0.47)	2.77 (0.50)
Respiratory exchange ratio, mean (SD)	1.06 (0.15)	1.06 (0.15)	1.08 (0.14)
Peak VO_2_ (mL/kg/min), mean (SD)	24 (8)	23 (8)	25 (9)

*Significantly (*p* < 0.05) more than the training cohort.

### 3.1 Agreement and association between manual and accelerometer-assessed step cadence

**Figure 1:**
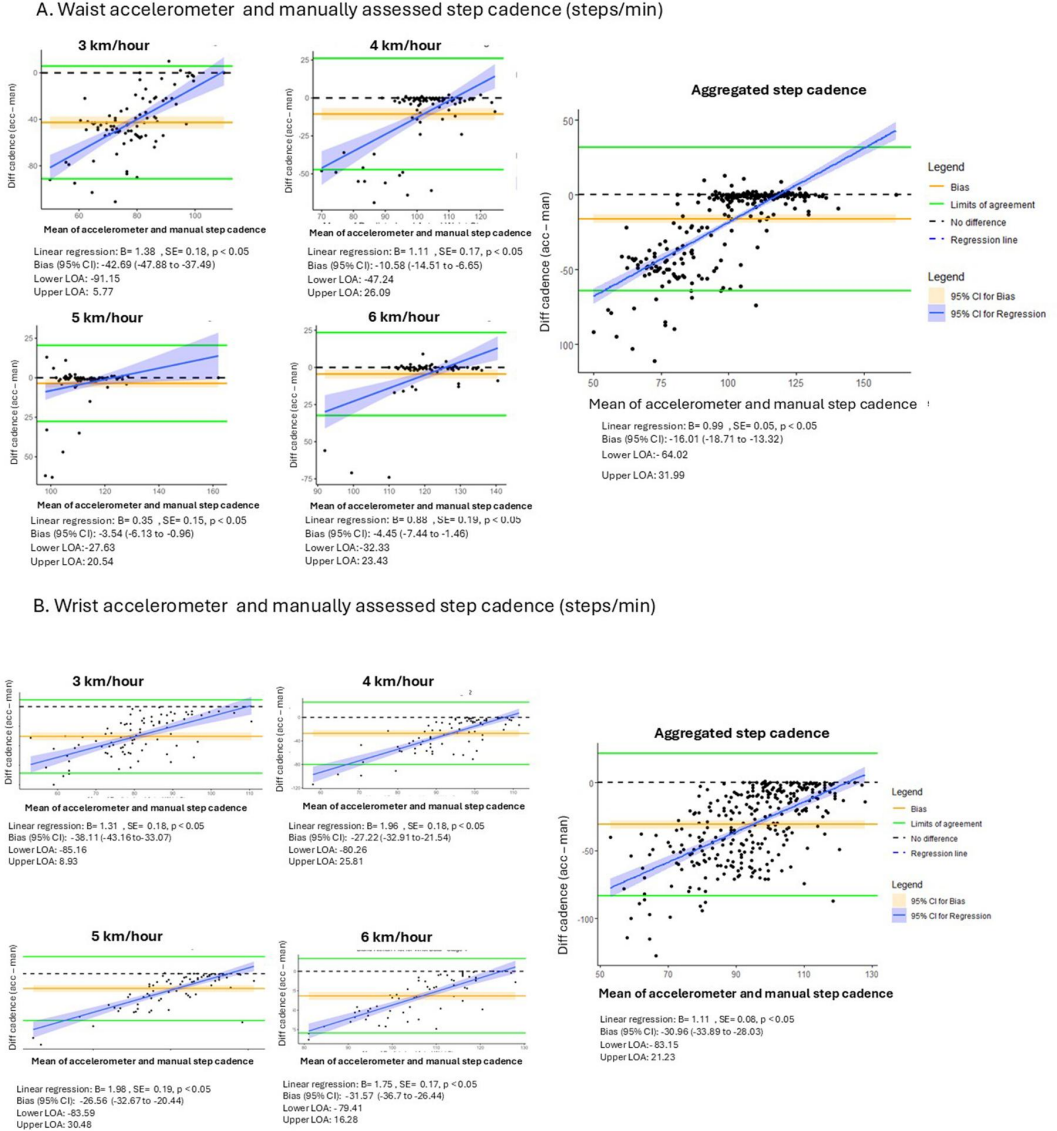
Bland–Altman plots for agreement of average manual and accelerometer (waist-worn **A**, wrist-worn **B**) step cadence. Figures present agreement in step cadence (steps/min) at 3, 4, 5, 6 km·h^−^^1^ and aggregated speeds. The solid blue line represents the crude mean difference between the two measures and the green lines are the crude 95% limits of agreement (LOA).

Median step cadence assessed manually and using waist- and wrist-worn accelerometers at different walking speeds, and aggregated values, is presented in Table 2. Bland–Altman analyses indicated that the step cadence for both waist and wrist accelerometer assessments was generally underestimated compared with the manual assessment (Figures 1A,B). At the lowest walking speed, mean differences were −43 and −38 steps/min for waist- and wrist-worn assessed accelerometer step cadence, respectively. At higher walking speeds, mean differences decreased to −4 and −5 steps/min for waist-worn and −26.56 and −31.57 steps/min for wrist-worn accelerometer assessment at 5 and 6 km·h^−^^1^, respectively. The aggregated waist- and wrist-worn accelerometer step cadence had mean differences of −16 and −33 steps/min, respectively. The dispersion of the differences (crude 95% limits of agreement) was wider at the lower step cadence. Linear regression analysis showed that higher average accelerometer step cadences were associated with smaller differences between the two assessments, reflecting stronger agreement (Figures 1A,B). Sensitivity analyses by age groups revealed that younger (<66 years) individuals had a lower mean difference compared with older individuals at 4, 5 km·h^−^^1^, and in aggregated accelerometer data relative to manual step cadence (*p* < 0.5). Similar results were seen for the wrist accelerometer step cadence, at 3, 4, 5 km·h^−^^1^, and aggregated data (Supplementary Appendix Table A2).

**Table 2:** Manual, waist- and wrist-worn accelerometer-assessed step cadence (steps/min) at four different speeds and aggregated.

Treadmill speed (km·h^−^^1^)	No. of observations	Percentage of VO_2_ peak, mean (SD)	METs, mean (SD)	Manually step cadence Med (q1–q3)	Accelerometer, waist Step cadence, Med (q1–q3)	Accelerometer, wrist step cadence Med (q1–q3)
3	87	47 (15)	3.62 (0.72)	99 (92.5–106)	53 (45–70.5)	59 (47.5–77)
4	85	53 (15)	4.11 (0.85)	106 (101–112)	102 (95–106)	86 (65–97)
5	81	61 (18)	4.96 (1.23)	113 (110–119)	111 (107–117)	96 (75–107)
6	69	70 (21)	6.11 (1.31)	121 (118–127)	120 (115–125)	96.5 (73–109)
Aggregated	216	na	na	111 (102–119)	105 (74–115)	79 (60–99)

### 3.2 Association between step cadence and absolute and relative cardiorespiratory intensity

Waist accelerometer step cadence at 3 km·h^−^^1^ and aggregated wrist accelerometer step cadence were excluded when exploring association with cardiorespiratory intensity due to low levels of agreement.

**Figure 2:**
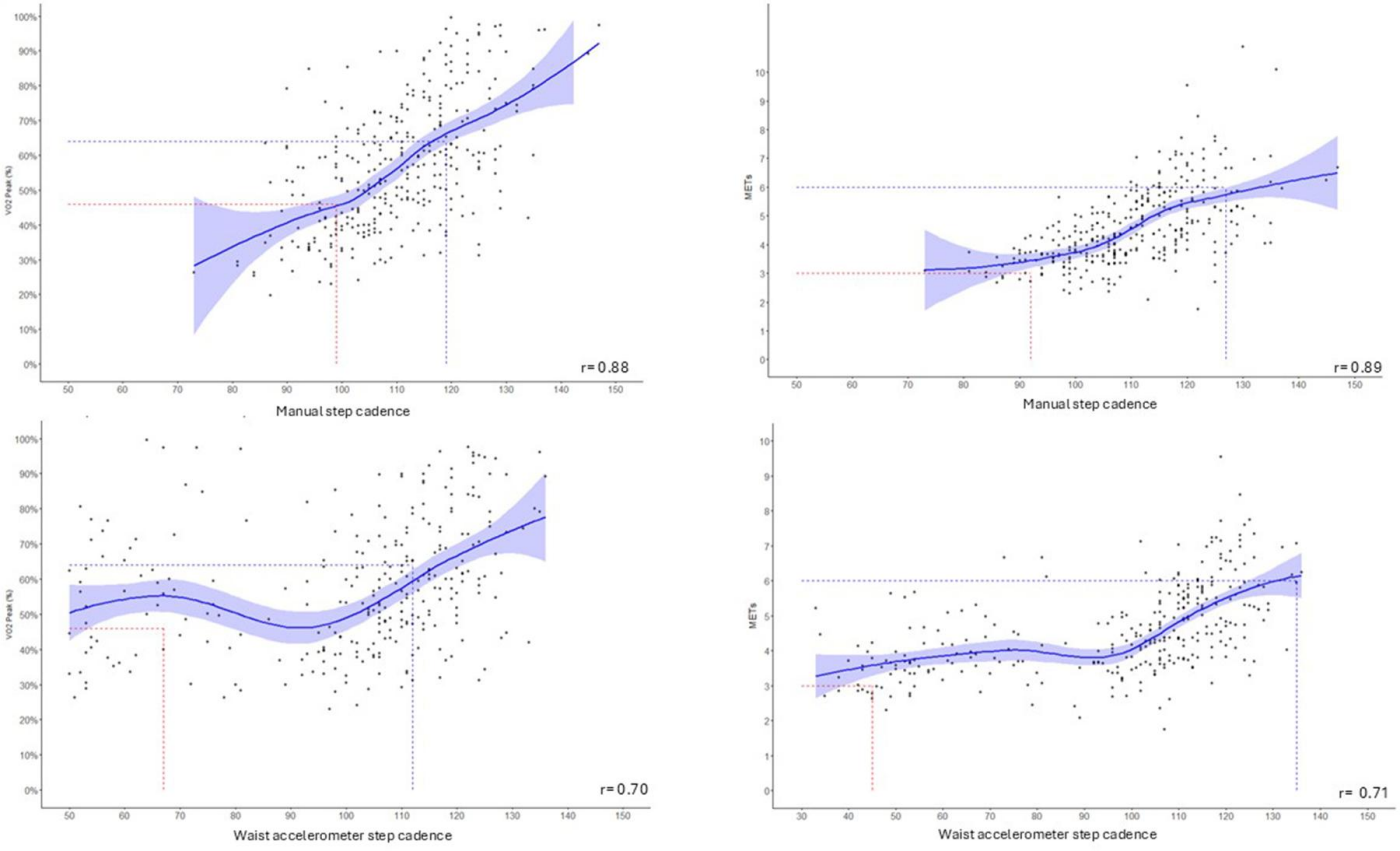
Association (with 95% CI) between step cadence (steps/min) and relative and absolute cardiorespiratory fitness. Dotted red lines illustrate moderate-intensity thresholds and blue dotted lines illustrate vigorous-intensity thresholds.

The correlation between step cadence and relative and absolute cardiorespiratory intensity was very strong (*r* = 0.9) for manual assessment and strong for waist-worn accelerometer assessment (*r* = 0.7) (Figure 2). The unadjusted linear GEE analyses revealed a significant and precise positive association between manual step cadence and relative (*β* = 0.0102, *p* < 0.001) as well as absolute (*β* = 0.08575, *p* < 0.05) cardiorespiratory intensity. Similar associations were observed for waist-worn accelerometer-assessed step cadence, with relative (*β* = 0.08575, *p* < 0.05) and absolute (*β* = 0.0335, *p* < 0.05) cardiorespiratory intensity. There was no substantial difference in the model when adjusting for sex, age, and BMI or height, with the step cadence coefficient remaining significant and QIC values comparable between adjusted and unadjusted models (Supplementary Appendices Tables A3, A4). In sensitivity analyses, inclusion of an interaction term between step cadence and age group showed a borderline significant result (*p* = 0.043) only for the model assessing absolute cardiorespiratory intensity with accelerometer-derived cadence. No significant interactions were observed in the other models, suggesting that the results were generally robust across age groups.

### 3.3 Step cadence thresholds

Table 3 presents step cadence thresholds for moderate and vigorous relative and absolute intensity developed in the training cohort. For manually assessed step cadence, the threshold for moderate relative intensity was 99 steps/min, while the step cadence associated with moderate absolute intensity was 92 steps/min. For vigorous intensity, manual step cadence thresholds were 117 and 127 steps/min for relative and absolute intensity, respectively. Waist-worn accelerometer thresholds for moderate intensity were 67 steps/min for relative and 45 steps/min for absolute intensity. For vigorous intensity, the thresholds were 112 and 135 steps/min for relative and absolute intensity, respectively.

**Table 3:** Step cadence (steps/min) thresholds, assessed manually and with a waist-worn accelerometer.

	Manual step cadence		Accelerometer waist step cadence	
	Relative intensity	Absolute intensity	Relative intensity	Absolute intensity
Moderate intensity				
Thresholds	99	92	61	45
Sensitivity	97%	95%	85%	98%
Specificity	39%	29%	25%	29%
PPV	79%	94%	72%	94%
NPV	85%	33%	41%	50%
Prevalence	70%	93%	70%	93%
Balanced accuracy	68%	62%	55%	63%
Vigorous intensity				
Thresholds	116	127	112	135
Sensitivity	67%	8%	56%	8%
Specificity	88%	94%	74%	100%
PPV	69%	17%	47%	100%
NPV	87%	86%	80%	87%
Prevalence	29%	14%	29%	14%
Balanced accuracy	77%	51%	65%	54%

PPV, positive predictive value; NPV, negative predictive value.

### 3.4 Validation of the developed accelerometer step cadence thresholds

Performance metrics across all thresholds in the validation cohort are described in Table 3. Manual step cadence thresholds demonstrated high sensitivity for detecting moderate relative and absolute intensity (97% and 95%, respectively), indicating that most moderate-intensity assessments were correctly identified. However, specificity was low (39% and 29%, respectively), suggesting that some low-intensity activity was incorrectly classified as moderate. Balanced accuracy was slightly higher for the relative intensity threshold (68%) than for the absolute intensity threshold (62%). For vigorous-intensity thresholds, sensitivity was lower, with markedly higher values for the relative intensity thresholds (67%) compared with absolute (8%). Meanwhile, specificity for both relative and absolute vigorous-intensity thresholds was strong, 88% and 94%, respectively, indicating that the threshold correctly identified individuals who reached vigorous cardiorespiratory intensity. Following the results for moderate thresholds, balanced accuracy was greater for the relative compared with absolute vigorous-intensity threshold (77% vs. 51%) (Table 3). PPV and NPV for absolute and relative intensity were consistent with the sensitivity and specificity findings (Table 3).

Validation values of the waist-worn accelerometer developed thresholds were in line with the results for the manually assessed step cadence thresholds. Sensitivity for detecting moderate cardiorespiratory intensity was high for both relative (85%) and absolute (98%) thresholds, while specificity for moderate intensity was lower at 25% and 29% for relative and absolute intensity, respectively. Balanced accuracy was lower for the relative intensity threshold (55%) than for the absolute (63%). For vigorous-intensity thresholds, sensitivity was low, with markedly higher values for the relative criterion (56%) compared with the absolute (8%), although specificity was high at 74% and 100% for relative and absolute intensity thresholds, respectively. The balanced accuracy was stronger for the relative than absolute intensity thresholds (65% vs. 54%) (Table 3). PPV and NPV for absolute and relative intensity were consistent with the observed sensitivity and specificity.

## 4 Discussion

The main findings of this study are as follows: (1) waist- and wrist-worn accelerometers tended to underestimate the step cadence compared with manual assessment, with stronger agreement at higher walking speeds and for the waist-worn devices. (2) There was a strong association between step cadence and cardiorespiratory intensity, with lower step cadence thresholds for moderate intensity (absolute and relative intensity) when assessed with a waist-worn accelerometer (45–61 steps/min) compared with manual assessment (92–99 steps/min). However, for vigorous intensity, there were small differences between accelerometer and manually assessed step cadence (steps/min) for both absolute (127 vs. 135) and relative (116 vs. 112) intensity.

This study indicates that step cadence for aggregated accelerometer assessment was underestimated for both waist (bias −16) and wrist (bias −31) compared with manual counts in people with CHD. This underestimation aligns with prior studies in the general population, regardless of activity monitor type or body placement (9–11). Agreement between accelerometer- and manually assessed cadence was lowest at 3 km·h^−^^1^ (−43 steps/min waist-worn; −38 steps/min wrist-worn). Similarly, Karac et al. observed mean differences of −121 steps and −49 steps per 2-min assessment for waist and left wrist accelerometers when walking 2 km·h^−^^1^ on the treadmill (10). In both our study and prior work (9–11), agreement strengthened as walking speed increased, with our study showing minimal differences at ≥5 km·h^−^^1^ (<−4 steps/min waist; <−32 steps/min wrist). Accelerometers may have less accuracy at low walking speeds, potentially due to the low acceleration amplitude being insufficient to consistently trigger step detection algorithms (22). Fokkema et al. reported that a walking speed of 3.2 km·h^−^^1^ was too slow for many participants to maintain a normal gait pattern, resulting in difficulties sustaining a constant walking pace, smaller step length, and minimal arm swing. The agreement of step cadence at all walking speeds (except for 3 km·h^−^^1^) was stronger with the waist-worn accelerometer than the wrist accelerometer in both this and other studies (9–11). This may be because accelerometers placed on the waist are close to the body's center of mass, aiding the device's accurate detection of whole-body acceleration (23). Importantly, we found a lower agreement among older individuals for both aggregated waist and wrist accelerometer assessments. The differences between age groups may be due to decreased gait stability with increased age (24), influencing accelerometer assessments.

The thresholds for moderate and vigorous intensity developed in this study varied between relative and absolute measures, reflecting the influence of individual fitness levels on step cadence. Our manually assessed threshold for relative moderate intensity (99 steps/min) was slightly lower than those reported by Tudor-Locke et al. in the general population (105–110 steps/min), possibly indicating that participants with CHD achieved comparable relative effort at lower step cadences. In contrast, thresholds for relative vigorous intensity were largely consistent with previous findings (115–120 steps/min) (15, 25). Similarly, our developed absolute thresholds showed a lower cadence for moderate activity (92 vs. 100 steps/min) but near equivalence for vigorous activity (127 vs. 130 steps/min) (13, 26).

Importantly, in this study, step cadence thresholds for moderate intensity were considerably lower when assessed using a waist-worn accelerometer compared with manual assessment, with thresholds of 45 steps/min for relative intensity and 61 steps/min for absolute intensity. Meanwhile, vigorous-intensity thresholds were similar across methods, with 112 and 135 steps/min for relative and absolute intensity, respectively. The improved alignment for vigorous intensity may be attributed to the stronger agreement between manual and waist accelerometer-assessed step cadence at higher walking speeds. The lower moderate-intensity thresholds identified using waist-worn accelerometers were consistent with those reported by Fridolfsson et al., who identified a threshold of 80 steps/min for moderate intensity in middle-aged adults in free-living conditions (12). Their lower thresholds from waist-worn accelerometers still exceeded ours, possibly due to their younger, healthier cohort without CHD. Although the thresholds derived from manual and accelerometer-based methods differed numerically, both demonstrated comparable sensitivity, specificity, and balanced accuracy for identifying absolute and relative moderate cardiorespiratory intensity. However, relative intensity step cadence thresholds seem to be superior compared with absolute intensity when developing cadence-based guidelines, especially for populations with diverse fitness levels.

To guide individuals with CHD, it is important to be aware of the methodological differences in step cadence thresholds, with a lower step cadence threshold for moderate intensity for accelerometer assessments. Importantly, these thresholds are lower compared with those developed for the general population, emphasizing that individuals with CHD may reach the recommendation of moderate intensity at a lower step cadence.

### 4.1 Strengths and limitations

To our knowledge, this is the first study to explore both the agreement between manual and accelerometer-assessed step cadence and the association between step cadence and relative and absolute cardiorespiratory intensity among individuals with CHD. The size of our study population is a strength, allowing stratification into different subgroups and validation of the developed thresholds in a validation cohort. Another strength is the use of GEE regression analyses, which provide a robust model for within-subject correlations across repeated measures (21). However, a limitation of this study is that it does not establish associations under free-living conditions, but rather in a laboratory environment. Finally, in line with previous studies among individuals with CHD (27), a large number of individuals did not reach an RER of ≥1.10, which may influence the results of relative intensity. Lastly, there were only a few female participants, decreasing the generalizability of the results for women, emphasizing the need for studies exploring this association among women.

## 5 Conclusion

Step cadence, whether assessed manually or via an accelerometer, is strongly associated with cardiorespiratory intensity and can effectively serve as a guide for physical activity recommendations in individuals with CHD. Although accelerometers tend to underestimate cadence at lower walking speeds, both methods demonstrate comparable accuracy in identifying intensity thresholds. Importantly, individuals with CHD may achieve moderate intensity at lower step cadences than the general population, based on assessments from both manual and waist-worn accelerometers.

## Data Availability

The raw data supporting the conclusions of this article will be made available by the authors, without undue reservation.
