# Supplementary material for: Step cadence to guide physical activity intensity in coronary heart disease
Source: Front Sports Act Living. 2026 Mar 16;8:1763343. doi: 10.3389/fspor.2026.1763343 (PMC13033787; doi:10.3389/fspor.2026.1763343)
Supplement: Supplementary file 1 [file Table1.docx]

Appendix Table A1, Participant characteristics, total cohort, and by mean age

| **Characteristic** | **Total cohort (n = 87)** | **Age < 66 years old** | **Age ≥ 66 years old** |
| --- | --- | --- | --- |
| Age, mean (SD) | 66 (10) | 57 (7) | 73 (5)* |
| Male, n (%) | 76 (87%) | 32 (84%) | 44 (90%)* |
| Height, mean (SD) | 1.74 (0.08) | 1.75 (0.08) | 1.73 (0.08) |
| Weight, mean (SD) | 83 (13) | 85 (11) | 81 (14) |
| BMI, median (IQR) | 27.5 (3.9) | 28.1 (3.9) | 27.0 (4.0) |
| Coronary treatment/intervention |  |  |  |
| Coronary artery bypass graft (CABG) | 17 (20%) | 7 (18%) | 10 (20%) |
| Conservative treatment | 8 (9.2%) | 4 (11%) | 4 (8.2%) |
| Percutaneous coronary intervention (PCI) | 62 (71%) | 27 (71%) | 35 (71% |
| Days from last cardiac event or interventon, median (IQR) | 515 (1,228) | 401 (999) | 789 (1,540) |
| Betablockers | 28 (35%) | 10 (30%) | 18 (38%) |
| Moderate to vigorous physical activity | 240 (270) | 210 (165) | 270 (280) |
| Resting metabolic rate, mean (SD) | 2.83 (0.47) | 2.90 (0.42) | 2.78 (0.50) |
| Respiratory Exchange Ratio, mean (SD) | 1.06 (0.15) | 1.10 (0.12) | 1.03 (0.16)* |
| Peak VO2, mean (SD) | 24 (8) | 28 (8) | 20 (6)* |
