# Supplementary material for: Step cadence to guide physical activity intensity in coronary heart disease
Source: Front Sports Act Living. 2026 Mar 16;8:1763343. doi: 10.3389/fspor.2026.1763343 (PMC13033787; doi:10.3389/fspor.2026.1763343)
Supplement: Supplementary file 2 [file Table2.docx]

Appendix Table A 2. Step cadence and Bland Altman results from manual and accelerometer-assessed step cadence (steps/min) across age groups

|  |  |  |  | | | |
| --- | --- | --- | --- | --- | --- | --- |
| **Age groups** | **Treadmill speed** | **Manual step cadence, median (Q1-Q3)** | **Accelerometer step cadence, median (Q1-Q3)** | **Bias (95% CI)** | **LoA,**  **lower-upper** | **B (SE)** |
|  |  |  | ***Waist*** |  |  |  |
| Younger individuals (<66 years) | 3 km·h⁻¹ | 100 (10) | 54 (21) | -46.98 (-54.03 to -39.92) | (-96.37 to 2.41) | 1.65 (0.25) |
| Older individuals (≥66 years) | 3 km·h⁻¹ | 95 (14) | 53 (32.5) | -46.98 (-54.03 to -39.92) | (-96.37 to 2.41) | 1.65 (0.25) |
| Younger individuals (<66 years) | 4 km·h⁻¹ | 107 (10) | 102 (15) | -37.16 (-44.56 to -29.76) | (-82.76 to 8.44) | 1.18 (0.24) |
| Older individuals (≥66 years) | 4 km·h⁻¹ | 104.5 (9.5) | 100 (10) | -15.04 (-21.26 to -8.83)* | (-58.53 to 28.45) | 1.37 (0.22) |
| Younger individuals (<66 years) | 5 km·h⁻¹ | 115 (9.5) | 112 (10.25) | -5.05 (-8.62 to -1.49) | (-27.02 to 16.92) | 0.52 (0.2) |
| Older individuals (≥66 years) | 5 km·h⁻¹ | 111.5 (10) | 111 (8.75) | -6.09 (-10.39 to -1.8)* | (-36.15 to 23.96) | 1.12 (0.28) |
| Younger individuals (<66 years) | 6 km·h⁻¹ | 124.5 (9.75) | 123 (10.5) | -0.66 (-2.75 to 1.44) | (-13.57 to 12.25) | -0.05 (0.1) |
| Older individuals (≥66 years) | 6 km·h⁻¹ | 120 (6) | 119 (7.5) | -5.65 (-10.58 to -0.71) | (-40.18 to 28.89) | 1.11 (0.33) |
| Younger individuals (<66 years) | Aggregated | 108 (18) | 105 (49) | -3.29 (-6.48 to -0.1) | (-22.95 to 16.38) | 0.74 (0.18) |
| Older individuals (≥66 years) | Aggregated | 111 (16) | 106 (23) | -19.99 (-24.05 to -15.93)* | (-72.79 to 32.81) | 1.14 (0.07) |
|  |  |  | ***Wrist*** |  |  |  |
| Younger individuals (<66 years) | 3 km·h⁻¹ | 100 (10) | 58 (28) | -43.65 (-50.77 to -36.53)* | (-93.48 to 6.18) | 1.62 (0.24) |
| Older individuals (≥66 years) | 3 km·h⁻¹ | 95 (14) | 65.5 (30) | -30.97 (-37.39 to -24.55) | (-70.55 to 8.6) | 0.97 (0.23) |
| Younger individuals (<66 years) | 4 km·h⁻¹ | 107 (10) | 80 (36.5) | -34.36 (-42.77 to -25.95)* | (-93.25 to 24.53) | 2.04 (0.22) |
| Older individuals (≥66 years) | 4 km·h⁻¹ | 104.5 (9.5) | 94.5 (23.5) | -18.39 (-24.72 to -12.07) | (-57.36 to 20.57) | 1.48 (0.32) |
| Younger individuals (<66 years) | 5 km·h⁻¹ | 115 (9.5) | 91.5 (33.75) | -32.58 (-41.4 to -23.77)* | (-94.29 to 29.12) | 2.31 (0.18) |
| Older individuals (≥66 years) | 5 km·h⁻¹ | 111.5 (10) | 100 (28.5) | -19.74 (-27.63 to -11.84) | (-68.39 to 28.91) | 1.26 (0.38) |
| Younger individuals (<66 years) | 6 km·h⁻¹ | 124.5 (9.75) | 97 (34.5) | -32.07 (-39.04 to -25.09) | (-80.91 to 16.78) | 1.54 (0.26) |
| Older individuals (≥66 years) | 6 km·h⁻¹ | 120 (6) | 92 (39) | -31.14 (-38.88 to -23.41) | (-78.81 to 16.53) | 2.08 (0.23) |
| Younger individuals (<66 years) | Aggregated | 108 (18) | 78 (40) | -36.2 (-40.49 to -31.9)* | (-91.99 to 19.6) | 1.3 (0.1) |
| Older individuals (≥66 years) | Aggregated | 111 (16) | 86 (35) | -24.98 (-28.69 to -21.27) | (-70.12 to 20.16) | 0.79 (0.12) |
