# Supplementary material for: Step cadence to guide physical activity intensity in coronary heart disease
Source: Front Sports Act Living. 2026 Mar 16;8:1763343. doi: 10.3389/fspor.2026.1763343 (PMC13033787; doi:10.3389/fspor.2026.1763343)
Supplement: Supplementary file 3 [file Table3.docx]

Appendix Table A3. Unadjusted and adjusted GEE model for the association between step cadence and cardiorespiratory intensity

| **Model type** | **Intercept, estimate** | **Steps, estimate** | **Standard Error estimate, steps** | **p-value** | **Wald-test** | **QIC** |
| --- | --- | --- | --- | --- | --- | --- |
| ***Manually assessed steps*** | |  |  |  |  |  |
| *Unadjusted GEE model* |  |  |  |  |  |  |
| Relative Vo2 | -0.545055 | 0.010178 | *0.0007613* | *<0.001* | 46.13 | 11.3 |
| Absolute Vo2 | -4.91215 | 0.08575 | 0.00581 | *<0.001* | 218 | 285 |
| *Adjusted GEE ^a^* |  |  |  |  |  |  |
| Relative Vo2 | -1.0772 | 0.0089 | 0.00075 | *<0.001* | 143.38 | 14.6 |
| Absolute Vo2 | -5.71592 | 0.08779 | 0.00597 | *<0.001* | 215.98 | 277 |
| ***Accelerometer assessed steps*** | |  |  |  |  |  |
| *Unadjusted GEE model* |  |  |  |  |  |  |
| Relative Vo2 | 0.243782 | 0.08575 | 0.000692 | *<0.001* | 26.2 | 10.7 |
| Absolute Vo2 | 1.4823 | 0.0335 | 0.0072 | *<0.001* | 21.6 | 284 |
| *Adjusted GEE ^a^* |  |  |  |  |  |  |
| Relative Vo2 | 0.30872 | 0.00294 | 0.00105 | *0.005* | 7.82 | 14.8 |
| Absolute Vo2 | 0.18158 | 0.03425 | 0.0072 | *<0.001* | 22.66 | 278 |

a Adjusted for age, sex and BMI
