# Supplementary material for: Step cadence to guide physical activity intensity in coronary heart disease
Source: Front Sports Act Living. 2026 Mar 16;8:1763343. doi: 10.3389/fspor.2026.1763343 (PMC13033787; doi:10.3389/fspor.2026.1763343)
Supplement: Supplementary file 4 [file Table4.docx]

Appendix Table A4. Unadjusted and height-adjusted GEE models for the association between step cadence and cardiorespiratory intensity

| **Outcome** | **Predictor** | **Unadjusted β (SE)** | **Adjusted β (SE)** | **QIC (unadjusted model)** | | **QIC (adjusted model)** |
| --- | --- | --- | --- | --- | --- | --- |
| METs | accstepswaist | 0.0335 (0.0072) | 0.0324 (0.0072 | 291.14 | | 275.8 |
| METs | mansteps | 0.0858 (0.0058) | 0.0843 (0.0058) | 304.46 | | 260 |
| %VO2peak | accstepswaist | 0.0035 (0.0007) | 0.0034 (0.0007) | 10.47 | | 14.37 |
| %VO2peak | mansteps | 0.0102 (0.0008) | 0.0102 (0.0008) | 12.01 | | 15.25 |
|  |  |  |  |  |  | |
