# Supplementary material for: Step cadence to guide physical activity intensity in coronary heart disease
Source: Front Sports Act Living. 2026 Mar 16;8:1763343. doi: 10.3389/fspor.2026.1763343 (PMC13033787; doi:10.3389/fspor.2026.1763343)
Supplement: Supplementary file 5 [file Presentation1.pptx]

## Slide 1
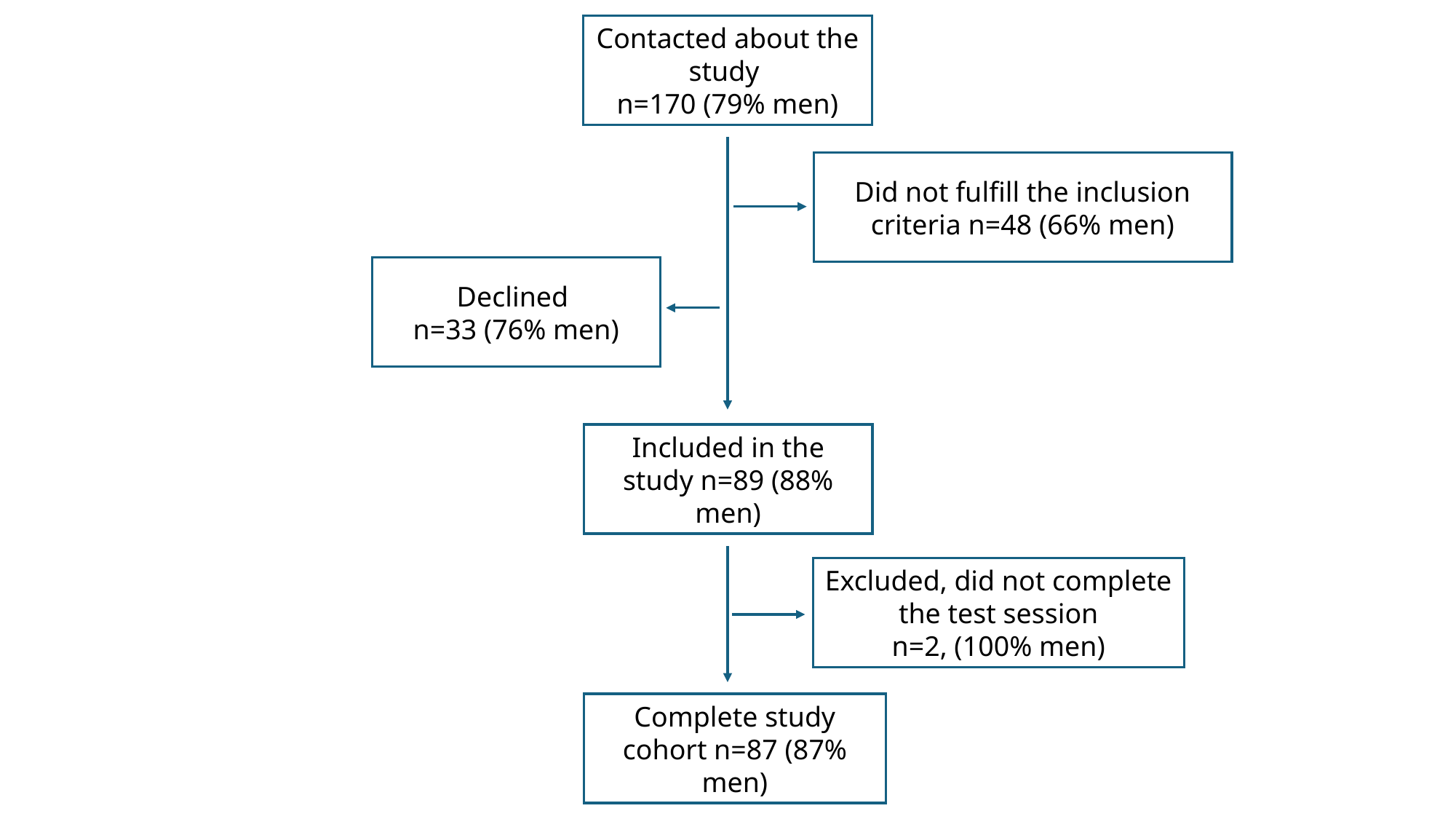

Contacted about the study
n=170 (79% men)
Did not fulfill the inclusion criteria n=48 (66% men)
Declined
n=33 (76% men)
Included in the study n=89 (88% men)
Excluded, did not complete the test session
n=2, (100% men)
Complete study cohort n=87 (87% men)
